# Delineating the conformational landscape and intrinsic properties of the angiotensin II type 2 receptor using a computational study

**DOI:** 10.1016/j.csbj.2022.05.012

**Published:** 2022-05-10

**Authors:** Xiaoliang Cong, Xiaogang Zhang, Xin Liang, Xinheng He, Yehua Tang, Xing Zheng, Shaoyong Lu, Jiayou Zhang, Ting Chen

**Affiliations:** a Department of Cardiology, Shanghai Changzheng Hospital, the Second Affiliated Hospital of Naval Medical University, Shanghai 200003, China; b Department of Cardiology, Shanghai University of Medicine & Health Sciences Affiliated Zhoupu Hospital, Shanghai 201318, China; c Medicinal Chemistry and Bioinformatics Centre, Shanghai Jiao Tong University, School of Medicine, Shanghai 200025, China; d Department of Cardiology, Changhai Hospital, Naval Medical University, Shanghai 200433, China

**Keywords:** G protein-coupled receptors, AT_2_R, MD simulations, Conformational dynamics, Cryptic pocket

## Abstract

As a key regulator for the renin-angiotensin system, a class A G protein-coupled receptor (GPCR), AngII type 2 receptor (AT_2_R), plays a pivotal role in the homeostasis of the cardiovascular system. Compared with other GPCRs, AT_2_R has a unique antagonist-bound conformation and its mechanism is still an enigma. Here, we applied combined dynamic and evolutional approaches to investigate the conformational space and intrinsic properties of AT_2_R. With molecular dynamic simulations, Markov State Models, and statistics coupled analysis, we captured the conformational landscape of AT_2_R and identified its uniquity from both dynamical and evolutional viewpoints. A cryptic pocket was also discovered in the intermediate state during conformation transitions. These findings offer a deeper understanding of the AT_2_R mechanism at an atomic level and provide hints for the design of novel AT_2_R modulators.

## Graphical abstract

**Figure:**
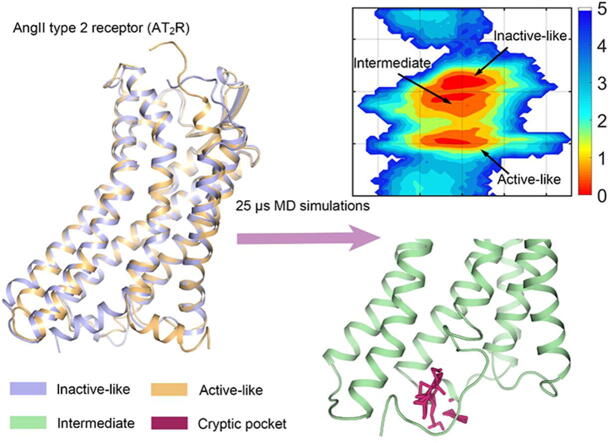

## 1 Introduction

The renin-angiotensin system (RAS) plays a critical role in maintaining the homeostasis of the cardiovascular system and its dysfunction leads to hypertension, heart disease and nephropathy [1], [2], [3], [4]. The main function of RAS is the direct regulation of the blood pressure via the octapeptide angiotensin II (AngII), whose receptors are two subtypes of class A G protein-coupled receptors (GPCRs), AngII type 1 receptor (AT_1_R) and AngII type 2 receptor (AT_2_R) [1], [5], [6]. Although sharing 34% sequence similarity, the two receptors show contrast function in cardiovascular system regulation, while the activation of AT_1_R increases the blood pressure, the activation of AT_2_R decreases it [7], [8], [9]. Moreover, AT_2_R has abundant functions such as inhibiting the cardiomyocytes autophagy [10], promoting vascular growth [11] causing anti-inflammatory effects [12], [13] and improving insulin resistance and metabolism [14], [15]. Thus, the regulation of AT_2_R is a promising field in new drug development and is capable of treating a number of pathological processes.

Normally, class A GPCRs have seven transmembrane (TM) helices linked by three extra- and three intracellular loops (ECLs, ICLs) with the helix 8 (H8) in the intracellular C-terminus [16], [17]. During its activation process, the endogenous AngII enters the orthosteric site located in the extracellular region of the TM bundle center. Then, the induced signal dynamically transfers through the TM domain and the cytoplasmic side of the activated receptor engages G proteins or β-arrestins [18], [19], [20]. The classical property for class A GPCR activation is the outward movement of TM6 helix [17]. However, AT_2_R is an outlier in the subfamily of GPCRs despite its similar sequence to AT_1_R. It influences the cellular activity via possible G protein, protein phosphatases and phospholipase pathways, but the affirmatory downstream protein has not been defined [8], [21], [22], [23], [24], [25].

Recently, its structures in both inactive-like, antagonist-bound and active-like, agonist-bound have become available [6], [21], [26]. In the antagonist-bound structure (PDB ID: 5UNG), its TM6 helix resembles the active conformation of other class A GPCRs but its helix 8 (H8) shows abnormal movement towards the center of the TM bundle [6]. Thus, it is regarded as an inactive-like state. The agonist-bound structure (PDB ID: 6JOD) shows an indistinct TM6 outward movement, while its H8 moves out from the TM bundle and become parallel with the membrane, like other class A GPCRs [26]. Because its ligand is the endogenous agonist angiotensin II, the structure is considered an active-like one. Although the static active-like, and inactive-like states of AT_2_R exhibit valuable structural divergences, it is still challenging to completely describe the conformational space of AT_2_R experimentally. Hence, it remains unclear how a dynamic pathway connects the two states of AT_2_R, thereby hindering the elucidation of how AT_2_R reaches the unique inactive state and the understanding of its activation and downstream signaling.

To dynamically unravel the GPCR conformational space, molecular dynamics (MD) simulations have been a well-established technique. With the application of MD simulations, the classical activation pathway for class A GPCR has been observed in β_2_ adrenergic receptor [27] and a cryptic pocket hidden in the metastable state of AT_1_R was discovered [28]. MD simulations have also captured precise molecule interplay with GPCRs (e.g. AT_1_R and GPR120) [29], [30]. To infer downstream signal protein in a dynamic way, MD simulations have also been applied to the A_2A_ adenosine receptor and μ-opioid receptor [31], [32]. Using statistical algorithms, such as Markov State Model (MSM), the detailed transition processes between states provide increasing insights into the activation [33], [34], [35], [36], [37].

Here, we used extensive all-atom MD simulations (25 μs) to depict the conformational landscape of AT_2_R and provided hints for its unique activation mechanism. Evolutional approaches were also referred to during our analysis. A hidden intermediate state was discovered, and it has a novel pocket for potential drug design. Our study not only sheds light on the research of AT_2_R mechanism but also provides an opportunity for the design of novel AT_2_R regulators.

## 2 Materials and methods

### 2.1 System setup

The AT_2_R structure in complex with an antagonist-like ligand, quinazolinone-biphenyltetrazole derivative 1 (compound 1 in [6], PDB ID: 5UNG), and structure complexed with the endogenous ligand, AngII (PDB ID: 6JOD), were downloaded from the Protein Data Bank (PDB). In the AngII bound structure, the ligand and co-crystallized antibody were removed. Meanwhile, the mutation on the structures was back-mutated referring to the wild-type sequence. The hydrogens were then added, while the termini were capped with acetyl and methylamine groups. The antagonist-bound complex was set as the inactive-like structure for simulations.

Compared with the antagonist-bound complex, the extra residues in AngII-bound structure were deleted to unify the atom connecting information for the following process. Then, we docked compound 1 to the apo AngII-bound structure using Molecular Operation Environment (MOE). After receptor preparation and minimization, ligands were initially placed in the pocket with the triangle matcher and London dG scoring. Next, rigid receptor with GBVI/WSA dG scoring was used for refinement. The output ligand pose with the smallest root mean square deviation (RMSD) with compound 1 in the antagonist-bound structure was picked for ligand coordinates for the active-like structure.

### 2.2 NEB sampling

To explore the conformational space of the AT_2_R, nudged elastic band (NEB) algorithm was first induced to generate initial structures on the free energy landscape. NEB determines the transition between different conformations by inserting a series of replicas between the initial and final states. The replicas have the same atom connection information as the inactive-like and active-like AT_2_R structures, but the coordinates are different from each other. The initial coordinates for each replica are evenly distributed along the pathway connecting the initial and final states. Then, elastic bands, a virtual spring force, were used to connect the replicas to their neighbors. With the elastic bands, structures can sample reasonable conformations in a simulated annealing process meanwhile avoiding sliding down to the energy basins [38]. The minimal energy pathway between the inactive-like and active-like AT_2_R structures was then described by the conformations of replicas.

The elastic bands were represented as 3 × N_atoms_ dimensional vectors [**R_0_**, **R_1_**, …., **R_n_**], which act on each atom to provide the restraint. **R_x_** means the replicas between the start state **R_0_** and end state **R_n_**. During calculation, a tangent vector **τ_i_** was induced to prevent the interference of elastic band forces and forces in MD force fields. Defined in Eq. (1), **τ_i_** controls the coordinates for each replica referring to the energy of this replica i (*V_i_*) and its neighbors.

(1) $$\tau i=\left\{\begin{array}{c} R_{i+1}-R_{i},V_{i+1}>V_{i}>V_{i-1}\\ R_{i}-R_{i-1},V_{i+1}<V_{i}<V_{i-1}\\ \left(R_{i+1}-R_{i}\right)\Delta V_{i}^{\max}+\left(R_{i}-R_{i-1}\right)\Delta V_{i}^{\min},V_{i}>V_{i+1}>V_{i-1}orV_{i+1}>V_{i-1}>V_{i}\\ \left(R_{i+1}-R_{i}\right)\Delta V_{i}^{\min}+\left(R_{i}-R_{i-1}\right)\Delta V_{i}^{\max},V_{i}>V_{i-1}>V_{i+1}orV_{i-1}>V_{i+1}>V_{i} \end{array}\right)$$

where$\Delta V_{i}^{\max}$ = max(|V_i+1_-V_i_|, |V_i−1_-V_i_|), $\Delta V_{i}^{\min}$= min(|V_i+1_-V_i_|,|V_i−1_-V_i_|).

With **τ_i_**, Eq. (2), (3) adjust the total force in its perpendicular ($F_{i}^{⊥}$) and parallel ($F_{i}^{\|})$ components, respectively.

(2) $$F_{i}^{⊥}=-\nabla V\left(Pi\right)+\left(\nabla V\left(Pi\right)Â\cdot \tau i\right)Â\cdot \tau i$$

(3) $$F_{i}^{\|}=\left(FsÂ\cdot \tau i\right)Â\cdot \tau i$$

(4) $$Ftotal=\;F_{i}^{⊥}{+F}_{i}^{\|}$$

where ∇V(*P_i_*) is the gradient of the potential energy according to the coordinates in replica i, namely the opposite of forces provided by MD force field. **F^s^** is the force from the elastic bands. Consequently, the force field and elastic bonds contribute to the perpendicular and parallel part of the total force and avoid interference between them [39], [40].

After the preparation, the active-like (PDB ID: 6JOD) and inactive-like (PDB ID: 5UNG) structures with antagonist-like ligand compound 1 were set as the initial and end states, respectively. The Amber ff19SB force field was employed for the description of atom interactions [41]. We firstly conducted 10,000 minimization cycles for our two systems. Then, 52 replicas were created between the initial and end structures.

During the NEB process, replicas were aligned to the center-of-mass and rotated by an optimal rotation matrix. The matrix minimizes the RMSD between structures in order to exclude the translational and rotational differences. In the simulated annealing process, the systems were firstly gradually heated to 300 K in 500 ps, with a spring force of 10 kcal∙mol^−1^∙Å^−2^. Then, replicas were equilibrated for 600 ps with a spring force of 50 kcal∙mol^−1^∙Å^−2^, which is kept in the coming processes. In the next simulated annealing runs, the system was generally heated to 500 K and cooled to 0 K in 1.5 ns. At last, the replicas were completely cooled at 0 K for 2 ns. The NEB workflow has been described and confirmed in the previous studies [28], [42].

### 2.3 MD simulations and analysis

To obtain different initial conformation in production MD simulations, we calculated the RMSD between adjacent replicas and picked the most different 10 replicas among 52 NEB outputs, including the active-like and inactive-like crystal structures. The following MD simulations are based on these structures.

The initial structures were inserted into a POPC (palmitoyl-2-oleoyl-*sn*-glycero-3-phosphocholine) membrane in the CHARMM-GUI server[43]. TIP3P waters with a length of 15 Å with 0.15 mol∙L^−1^ KCl were added to the top and bottom of the system. FF19SB, LIPID17, and GAFF2 force field were applied for the parameter of amino acids, lipids, and ligand, respectively [41], [44], [45]. The components of bilayers have been commonly used in other simulations[27], [46], [47], [48].

The systems were firstly minimized for 15,000 cycles with a restraint of 500 kcal∙mol^−1^∙Å^−2^ on the protein and lipids. Then, all atoms encountered 30,000 cycles of minimization. Next, the systems were heated from 0 to 300 K in 300 ps and equilibrated for 700 ps with 10 kcal∙mol^−1^∙Å^−2^ position restraint on non-solvent atoms. At last, the 10 systems encountered 5 rounds of 500 ns production MD simulations, leading to 50 independent repeat trajectories of 25 μs in total. During simulations, the temperature (300 K) and pressure (1 atm) were controlled by the Langevin thermostat and Berendsen barostat, respectively. Long-range electrostatic interactions were treated by the Particle mesh Ewald algorithm and a cutoff of 10 Å was employed for short-range electrostatic and van der Waals interactions. The SHAKE algorithm was applied to restrain the bond with hydrogens. for covalent bonds containing hydrogen. All simulations were finished on Amber20, pmemd.cuda on NVIDIA Tesla V100 PCIe 16 GB.

The analyses were accomplished by Amber20 CPPTRAJ [49]. In particular, RMSF was calculated by the “atomicfluct” command, PCA was calculated by “projection” commands, distance and dihedral were calculated by “distance” and “dihedral” commands, and DCCM was calculated by the “matrix” command. Figures were drawn by MATLAB and PyMOL.

### 2.4 Statistics coupled analysis (SCA)

Multiple sequence alignment (MSA) of AT_2_R was based on the alignment from the GPCRdb database [50]. All source and all species available AT_2_R sequences were used. The sequences were then adjusted to fit the sequences of 6JOD. In total, 121 sequences and a row of gaps to prevent the zero-frequency problem were set as input for SCA [51].

In the SCA process, the conservation score for position i with amino acid a (D_i_^a^) was estimated by the cross-entropy loss between actual a frequency at the position (f_i_^a^) and background frequency for this residue (q^a^). The loss was calculated in Eq. (5).

(5) $$D_{i}^{a}=f_{i}^{a}\times \ln\left(f_{i}^{a}/q^{a}\right)+\left(1-f_{i}^{a}\right)\times \ln\left(\left(1-f_{i}^{a}\right)/\left(1-q^{a}\right)\right)$$

In each position, the most dominant amino acid was picked to calculate the frequency f_i_^a^. Then, the correlations between positions i and j with the dominant residue a and b ($C_{\mathrm{ij}}^{\mathrm{ab}}$), namely the co-evolution score, was estimated by Eq. (6).

(6) $$C_{\mathrm{ij}}^{\mathrm{ab}}\;=\;\frac{\partial D_{i}^{a}}{\partial f_{i}^{a}}\;Â\cdot \;\frac{\partial D_{j}^{b}}{\partial f_{j}^{b}}\;Â\cdot \left|f_{\mathrm{ij}}^{\mathrm{ab}}-f_{i}^{a}Â\cdot f_{j}^{b}\right|$$

where $f_{\mathrm{ij}}^{\mathrm{ab}}$ represents the frequency that position i has residue a as the dominant one, meanwhile position j has residue b as the dominant one. $C_{\mathrm{ij}}^{\mathrm{ab}}$ values constitute the SCA matrix.

The eigenvectors (principal components, PC) of the SCA matrix were next calculated to identify the evolutional related positions (sectors). As the first mode reflects the global fluctuations during the evolution process, it was dropped in the definition of sectors. In the sector definition, different sectors are divided clearly on the PC surface and have limited evolutional relationships intra-sectors. Thus, red sectors are defined as the positions whose weight of the 2nd eigenvector is larger than the weight of the 4th eigenvector and larger than ε. Blue sectors are the positions whose weight of the 2nd eigenvector is smaller than the weight of the 4th eigenvector and -ε, or whose weight of the 4th eigenvector is larger than the weight of the 2nd eigenvector and larger than ε. In the application of AT_2_R, the threshold value ε was 0.05. The sector selection and SCA calculation referred to previous publications [51], [52].

### 2.5 Markov state Model (MSM) construction

According to the activation parameters, an MSM was built using the Python Emma's Markov Model Algorithms (PyEMMA) package [53]. Firstly, the points in the free energy landscape were clustered into 500 microstates by the k-means algorithm. Then, multiple transition probability matrixes (TPMs) were calculated according to the transitions among microstates. Referring to Eq. (7), the implied timescale test was performed to confirm the Markovian of microstates.

(7) $$\tau_{i}=-\tau /\ln\lambda_{i}$$

where *τ* represents the lag time for the TPMs, *λ_i_* is the i^th^ eigenvalue of the TPM and *τ_i_* is the implied timescale for the i^th^ relaxation of the MSM. As a function of the lag time τ, *τ_i_* (especially τ^1^ for the slowest transition) is a constant when the transition between microstates is Markovian [33], [54]. As shown in Fig. 5C, the lagtime for MSM construction was 4 ns. From Markovian microstates, macrostates were clustered via the PCCA + algorithm. Using transition path theory (TPT), the properties for transition, such as transition time and direction, were calculated [55]. To obtain the representative structures, the snapshots around the microstate centers of each macrostate were extracted to a trajectory. Then, the representative conformation of each macrostate was picked according to the similarity score *S_ij_* estimated via Eq. (8).

(8) $$S_{\mathrm{ij}}=e^{-dij/dscale}$$

where the *d_ij_* is the RMSD between the snapshots i and j and *d_scale_* is the standard deviation of *d*.

### 2.6 Pocket identification and molecule docking

Using Fpocket, we identified the potential pockets of different macrostates[56]. Fpocket defines a sphere that contacts four atoms on its boundary and contains no internal atom as an alpha sphere. It then selects alpha spheres defined by zones of tight atom packing. In the following cluster step, it excludes large spheres at the protein surface which solely composes a sphere cluster, then aggregates clusters with a close center of mass to a large cluster. Next, multiple linkage clustering approaches are used to further merge clusters. At last, the ability to bind a small molecule was evaluated by Partial Least Squares fitting to pocket descriptors, and top-scored pockets are shown.

From the pocket, we also conducted molecular screening using the allosteric GPCR sublibrary of Enamine. During the screening, all compounds were firstly prepared at pH 7.0 with the OPLS3 force field. Then, docking in standard precision was applied for all compounds Then, 1,000 top-scored compounds encountered extra precision docking to produce the final output. The docking procedure was performed by glide in Maestro, Schrödinger suites.

## 3 Results

### 3.1 Large-scale unbiased MD simulations unravel the conformational space of AT_2_R.

**Fig. 1:**
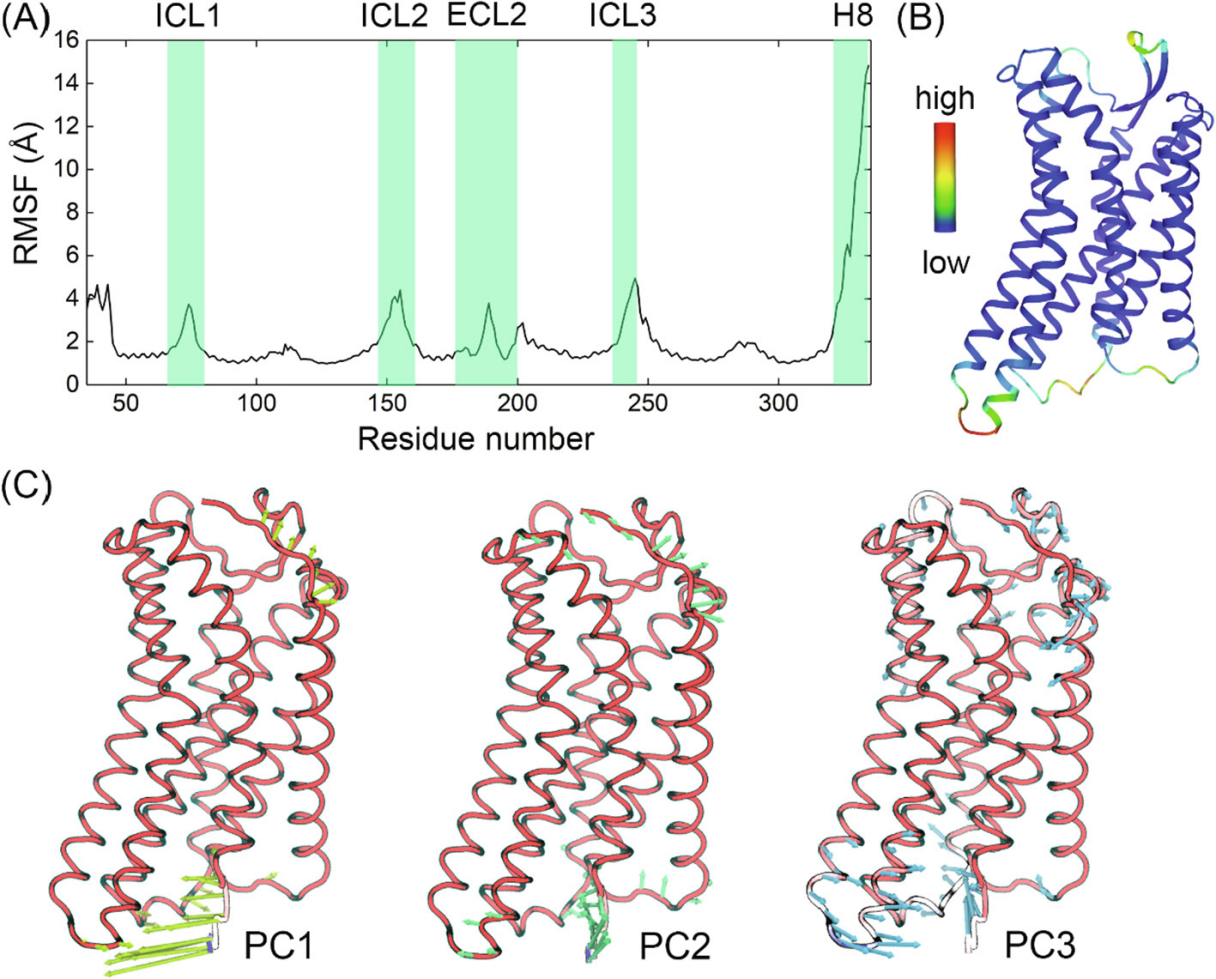
(A) RMSF value for each residue during MD simulations. Flexible domains are highlighted in green rectangles. (B) The structure colored by the RMSF value. Highly flexible domains such as N-term and H8 were removed for clarity. (C) The movement expressed by major PCs. Yellow, green, and blue arrows represent the movement shown by PC1, PC2, and PC3, respectively. (For interpretation of the references to color in this figure legend, the reader is referred to the web version of this article.)

To sample the conformational landscape of AT_2_R and understand its transition pathway towards the inactive-like conformation, we first generated a series of replicas connecting the inactive-like (PDB ID: 5UNG) and active-like (PDB ID: 6JOD) crystal structures with agonist bound. Then, nudged elastic band algorithm was applied to sample 10 initial structures distributed on the transition pathway. The structures were embedded in the POPC membrane and encountered 500 ns*5 independent MD simulation runs, leading to a total simulation time of 25 μs. With the trajectories, we first calculated the root-mean square fluctuation (RMSF) of each residue (Fig. 1A and B) and decomposed the movements shown in the principal component analysis (PCA) to residues (Fig. 1C), to determine the flexible domains and their intrinsic movement patterns.

As shown in Fig. 1A, H8 is the most flexible domain during simulations. Besides H8, the intracellular loops are all flexible and ECL2 also shows obvious movement. According to the RMSF values shown in Fig. 1B, ICL1 and ICL2 are only flexible in the loop domain but ICL3 leads the movement for close intracellular TMs. As for the decomposition of movement shown in PCA (Fig. 1C), the dominant movement comes from H8 while the movement of intracellular TM5-TM6 is also evident in PC3. However, other domains with high RMSF show no obvious movement tendency in the decomposition of PCA. Thus, the major movements in AT_2_R conformation transition were the movement of H8 and the intracellular TM5-TM6. Since H8 conformation is distinctive in the start and end structures of NEB, it is expected to show highly dynamic but the in-detail transition process is still worth investigation.

**Fig. 2:**
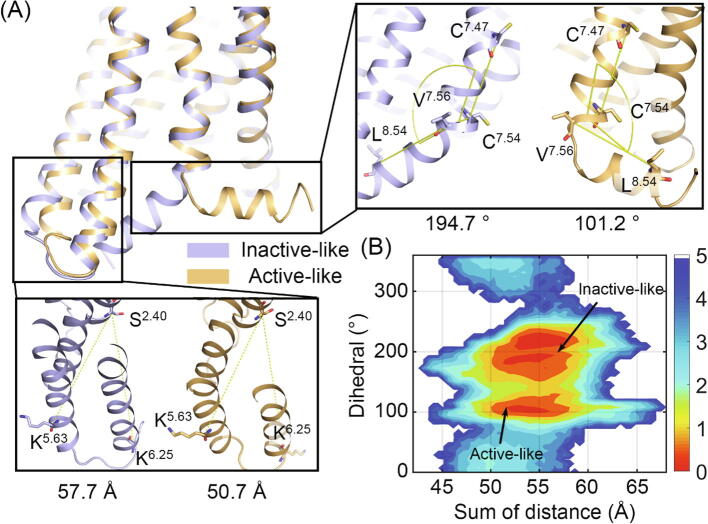
(A) The distinctions between the AT_2_R in the two states. Purple and orange cartoons show inactive-like and active-like AT_2_R, respectively. In the zoom-in views, the sum of distance between the Cα atoms of S^2.40^ to K^5.63^ and S^2.40^ to K^6.25^ was shown to measure the movement of TM5-TM6. The dihedral among the Cα atoms of L^8.54^, V^7.56^, C^7.54^, and C^7.47^ was applied for the movement of H8. (B) The free energy landscape composed of the sum of distance and dihedral. Arrows show the position of inactive-like and active-like AT_2_R on the landscape. (For interpretation of the references to color in this figure legend, the reader is referred to the web version of this article.)

Given that the common transducer pocket is composed of TM5-TM6 and H8 [17], [28] and their aforementioned dominant movement, we defined the conformation-describing parameter for AT_2_R according to the movement of intracellular TM5-TM6 and H8 (Fig. 2A). Since TM2 is relatively stable, the sum of the distance from K^5.63^ to S^2.40^ and K^6.25^ to S^2.40^ reflects the movement of intracellular TM5-TM6 (superscripts indicate the Ballesteros–Weinstein numbering system [57]). In addition, the fluctuation of H8 in AT_2_R covers the space from the center of the TM bundle to the position in parallel with the membrane, which is described by the dihedral among L^8.54^, V^7.56^, C^7.54^, and C^7.47^. As shown in Fig. 2A, the distance and dihedral values are distinctive between the inactive-like and active-like states, thereby confirming their power to depict the difference in different AT_2_R states.

With the conformation-describing parameters, the conformational space of AT_2_R during MD simulations was plotted as a free energy landscape in Fig. 2B. The active-like structure (50.7 Å, 101.2°) locates at an energy basin with a distance of ∼52–56 Å and a dihedral of ∼100–110°. The fixed dihedral indicates that H8 paralleled with the membrane is stable, maybe attributing to the interaction with membrane parts. This H8 conformation is also widely adopted by other class A GPCRs [17], [58]. Upon the increasing of dihedral from active-like basin, the sum of distance also increases to 54–57 Å, in order to cross the energy barrier between a dihedral of ∼130–150°. It infers that the movement of H8 couples with the open degree of intracellular TM5 and TM6. From a dihedral of around 150°, the relative energy of conformations becomes lower and two different energy basins (∼51–56 Å and 180-197°, ∼53–58 Å and 203v230°) locates there. Situated at (57.7 Å, 194.7°), the inactive-like structure locates at the low energy area and the specific basin for the inactive-like state will be identified in the coming parts. From a single trajectory, a small number of points are out of major conformations at a dihedral of 80–250°, reflecting a disordered H8. The isolated phenomenon should be excluded from the main conformation change. Collectively, the free energy landscape shows the conformational space of AT_2_R on its intracellular side and illustrates the intrinsic dynamic of AT_2_R.

### 3.2 Evolutional and dynamical analysis reveals the relationship between domains in signal transportation of AT_2_R

**Fig. 3:**
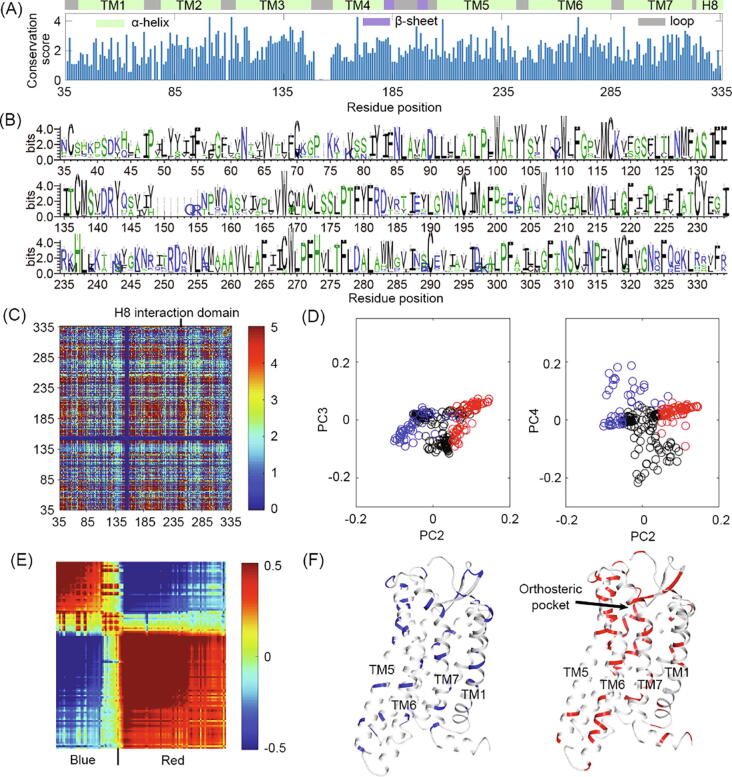
(A) The conservation score of each position in a multiple sequence alignment for AT_2_R sequences. Secondary structures for positions were labelled above. Color scheme: green, α-helix; purple, β-sheet; gray, loop. (B) Logoplots visualizing residue frequency in each position. (C) SCA matrix for residue pairs. From blue to red, the evolutional correlation increases. H8 interaction domain is labelled by a line. (D) The projection of residues on the PC2-PC3 and PC2-PC4 decomposition for SCA matrix. Blue and red circles identify corresponding sector residues. (E) The clustered co-evolution matrix for blue and red sectors. (F) The location of blue and red evolutional sectors on AT_2_R. Sector residues are colored correspondingly and the other residues are shown in gray cartoons. Key helixes and position of the orthosteric pocket are labelled. (For interpretation of the references to color in this figure legend, the reader is referred to the web version of this article.)

To unravel the relationship between intracellular movement and orthosteric pocket, we applied evolutional and dynamical algorithms on AT_2_R. Statistics coupled analysis (SCA) is an approach to estimating the co-evolution degree between residue pairs, thereby identifying the evolutional- and functional-related residue sectors according to the degree [51], [52], [59]. We used all sequences of AT_2_R from different species (see methods) and calculated the conservation and co-evolution property in each position (Fig. 3). The conservation score and logoplots for each position are shown in Fig. 3A, 3B, respectively. Also, Table 1 summarizes the conservation score for each domain. The co-evolution score between each residue pair forms the SCA matrix in Fig. 3C. Then, the evolutional-related sectors were identified by eigenvector decomposition (Fig. 3D). The red and blue sectors have a clustered co-evolution matrix in Fig. 3E, while their positions are shown in Fig. 3F.

**Table 1:** Average conservation score for different AT_2_R domains.

Domain name	Residue range	Average conservation score
N-terminal	35–44	1.52 ± 0.57
TM1	45–72	1.69 ± 0.83
TM2	78–107	2.28 ± 0.78
TM3	112–148	2.31 ± 0.77
TM4	156–182	2.15 ± 0.69
TM5	203–241	2.12 ± 0.78
TM6	245–285	2.24 ± 0.72
TM7	289–321	2.13 ± 0.81
H8	322–335	1.70 ± 0.73
ICLs	73–77, 149–155, 242–244	0.59 ± 0.86
ECLs	108–112, 183–202, 286–288	2.03 ± 0.82
All loops		1.53 ± 1.08
All TMD		2.12 ± 0.78

As shown in Fig. 3A and Table 1, the conservation degree is different among domains. TMD (score = 2.12 ± 0.78) is more conserved than loops (score = 1.53 ± 1.08), especially for the ICLs (score = 0.59 ± 0.86). Since ECLs are the gate for the orthosteric pocket [60], its conservation (score = 2.03 ± 0.82) infers that AT_2_R changes its ligand binding process a little during the evolution process. In contrast, ICLs traditionally interact with downstream proteins and the variation of ICLs reflects possible distinct AT_2_R-interacting proteins (ATIP) in species [61], [62]. Variation in conservation is not obvious in TM2-TM7 but TM1 (score = 1.69 ± 0.83) and H8 (score = 1.70 ± 0.73) are not as conserved as other TM helixes, inferring that TM1 and H8 may play unique roles in different AT_2_Rs.

The logoplots in Fig. 3B correspond with the conservation score in Fig. 3A and directly show the most conserved sequence in AT_2_R. Globally, most positions have mutations and some of them are even highly mixed (e.g. 64, 119, 169, and 243), which provides information for the following co-evolution analysis. Also, key activation motifs for class A GPCRs, such as DR142^3.50^Y, P223^5.50^-I132^3.40^-F265^6.44^, CWxP271^6.50^, and NP315^7.50^xxY are still conserved in AT_2_R logoplots. It suggests that AT_2_R maintains a traditional signal pathway in class A GPCRs.

In Fig. 3C, the spots with the most co-evolution scores to other domains are N156^4.38^ to G210^5.37^, L239^5.66^ to T250^5.29^, and G285^6.64^ to L298^7.33^, which represent the extracellular TM4-TM5, intracellular TM5-TM6, and extracellular TM6-TM7, respectively. We also identified that H8 has higher co-evolution scores with the H8 interaction domain in [6] than other residues, as labeled in Fig. 3C. For instance, R324^8.49^ has a high co-evolution score with directly interacting residues Q253^6.32^ (2.87) and M257^6.26^ (2.84). However, for other residues around, the average co-evolution score is 0.70 ± 0.64. It confirms that the co-evolution score reflects interactions between domains.

In Fig. 3D, using PCA, we decomposed the SCA matrix to PC2-PC4 and defined evolutional related blue and red sectors (see method). In the clustered SCA matrix (Fig. 3E), residues in blue and red sectors show limited intra-action but evident interaction, confirming the separation of sectors. As shown in Fig. 3F, the blue and red sectors include the residues with the most co-evolution scores. The blue sector is mostly on the membrane side, while the red sector consists of residues around the orthosteric pocket and intracellular TM5-TM6. Evolutional related sectors are spatial and functional closed residues [52]. Thus, considering their position, the blue sector may contribute to the stability of AT_2_R in the membrane, while the red sector shows the relationship between the ligand and classical transducer pockets. Since the conserved motifs are also maintained in AT_2_R sequences, the original signal pathway may still exist in AT_2_R, though it is controversial to interact with classical transducer proteins.

**Fig. 4:**
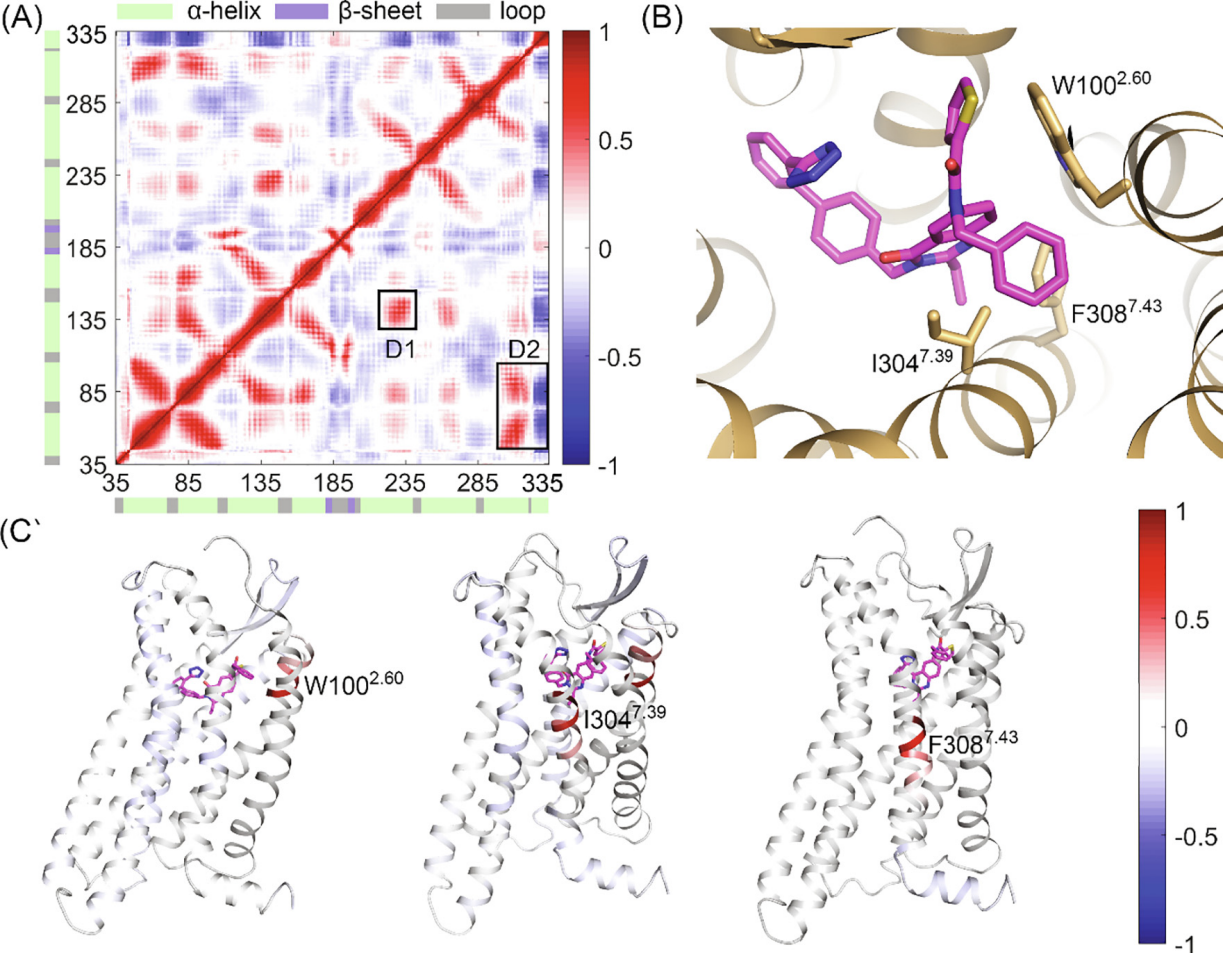
(A) The DCCM matrix between residue pairs. Red and blue show correlated and anti-correlated movement between the pair, respectively. The correlation with a constant smaller than 0.3 was colored white for clarity. Rectangles show key correlation domains. Secondary structures of residues are shown around the residue index. Color scheme: green, α-helix; purple, β-sheet; gray, loop. (B) The position of highly contacting residues (W100^2.60^, I304^7.39^, and F308^7.43^, more than 60% in contact with ligand at the threshold of 4 Å) and ligand. AT_2_R and ligand were colored orange and magentas, respectively. (C) The projection of DCCM score for highly contacting residues on AT_2_R structure. The color scheme is the same as (A). (For interpretation of the references to color in this figure legend, the reader is referred to the web version of this article.)

The dynamic cross correlation matrix (DCCM) was also applied to show the relationship of movement between residue pairs. DCCM algorithm estimates the correlation between residue movement, which is shown as a DCCM matrix shown in Fig. 4C [63]. Red and blue in the matrix reflect correlated and anti-correlated movements, respectively. To explore the correlation between the orthosteric pocket and intracellular part for conformation-describing parameters, we first identified residues highly contacting with ligand in the pocket (Fig. 4B). Then, the DCCM matrix for these residues with other residues was visualized in Fig. 4C.

From the global DCCM matrix, the intracellular TM5 (A228^5.55^ to T241^5.68^) mostly correlates with TM3 (D1, A130^3.38^ to Q144^3.52^), while the intracellular TM6 (G245^6.24^ to K256^6.35^) has no obvious interaction with other residues. As for the H8 (G322^8.47^ to R334^8.59^), its movement is mostly related to TM1, ICL1, and TM2 (D2, D45^1.33^ to V88^2.48^), which may be attributed to their close position. In particular, the highly-contacting residues in Fig. 4B show a weak movement relationship with classical activation helices (W100^2.60^ with TM6 and TM7, I304^7.39^ with TM6 and H8, and F308^7.43^ with H8). Since the transferring of a signal from orthosteric pocket to TM6-H8 is the base of class A GPCR activation [17], the relationship between highly contacting residues and TM6-H8 infers that a typical signal pathway still exists in AT_2_R. In total, evolutional and dynamical analysis shows the uniquity of AT_2_R, but the classical GPCR signal pathway is still maintained.

### 3.3 Markov state model discovers distinct dynamic properties of AT_2_R

**Fig. 5:**
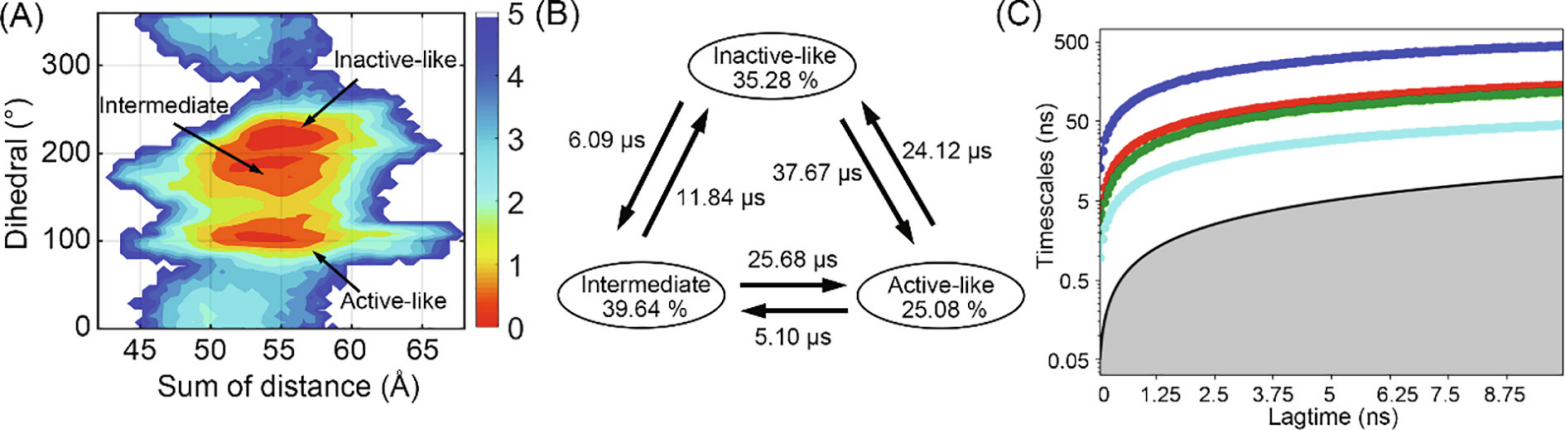
(A) The position of macrostates on the free energy landscape and their corresponding states in activation. (B) The transition between macrostates and proportions of each state. (C) The implied timescale test for the MSM. Different timescales τ_1_, τ_2_, τ_3_, and τ_4_ were represented as blue, red, green, and cyan lines changing with lag time. (For interpretation of the references to color in this figure legend, the reader is referred to the web version of this article.)

**Fig. 6:**
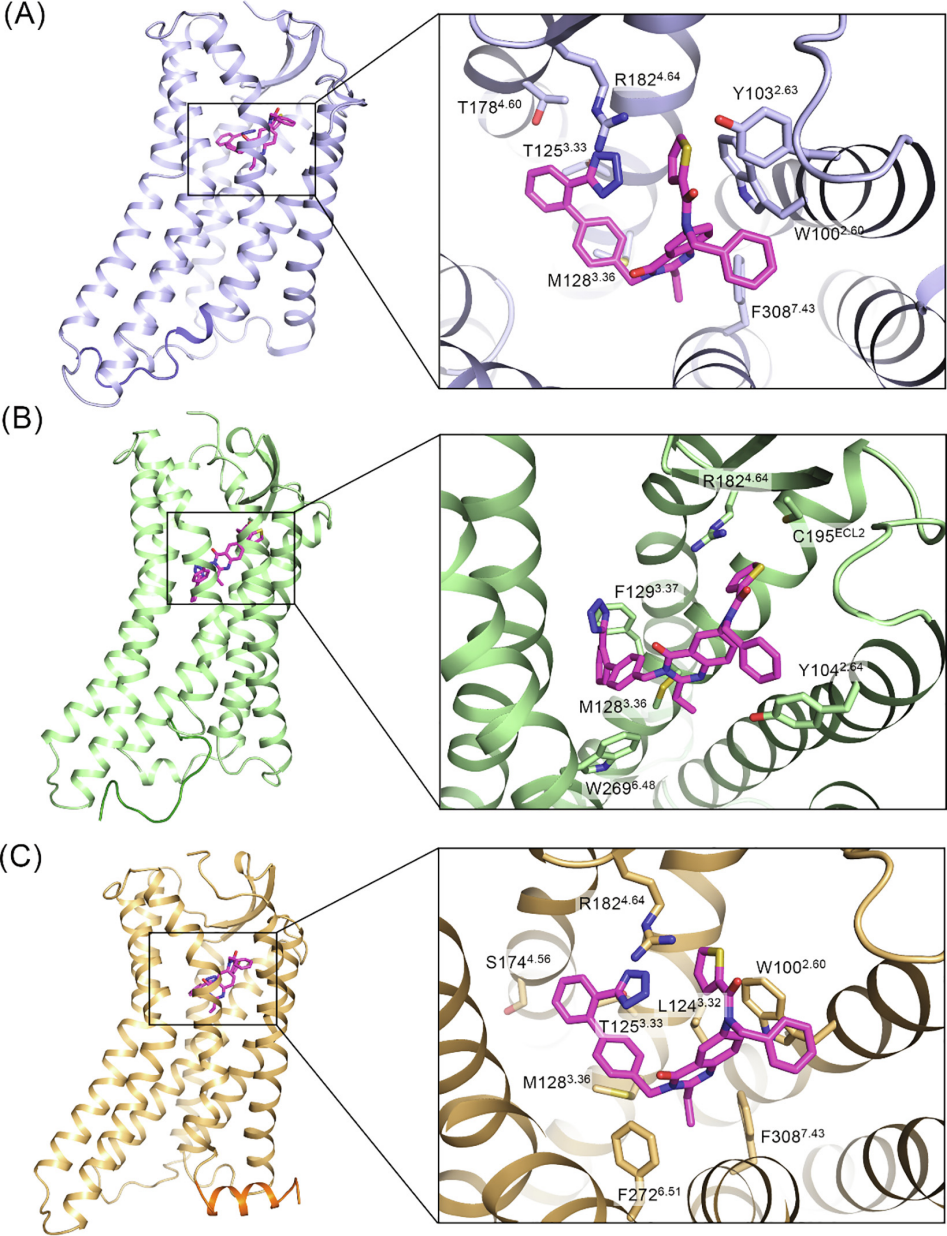
The representative structure and pocket conformation for inactive-like (A), intermediate (B), and active-like (C) AT_2_R, which are shown in purple, green, and orange cartoons. The dark color identifies the position of H8. The interface residues and ligand are shown in sticks. (For interpretation of the references to color in this figure legend, the reader is referred to the web version of this article.)

To describe the detailed movement of the intracellular domain and further explain the free energy landscape, we built a kinetic network MSM with the conformation-describing parameters. Using the estimation of transition between different states, MSM offers a statistical viewpoint for the conformational ensemble during MD simulations [33]. Three macrostates were clustered from MSM (Fig. 5A) and the transitions between them are shown in Fig. 5B. The activation state of each macrostate is determined via their corresponding representative structure. The result of the implied timescale test in Fig. 5C shows a flattened curve from 4 ns in all timescales. Thus, the choice of 4 ns as lagtime confirms the Markovian for our model. We also show the representative structure for each macrostate in Fig. 6.

Since our systems include an antagonist, the transition in Fig. 5B does not favor active-like structure. The active-like structure is easy to transfer to the intermediate state (5.10 μs), but it would be difficult to go reversely (25.68 μs). Although no direct pathway exists between active-like and inactive-like states, the transition time (24.12 μs to inactive-like and 37.67 μs to active-like) reflects the effect of the antagonist. The proportion of active-like state is, therefore, the least (25.08%). Notably, the proportion of intermediate (39.64%) and inactive-like (35.28%) states are similar, even the transition between the two states leans towards the intermediate state. It infers that the antagonist also permits the existence of the intermediate state. Considering a 500 ns timescale for each trajectory, the proportions may include many snapshots during the transition, so the specific values are for reference.

As the inactive-like crystal structure locates at (57.7 Å, 194.7°), the inactive-like macrostate shows a larger H8 dihedral (more than 200°), suggesting a conformation closer to the TM bundle center (Fig. 5A and Fig. 6A). The intermediate macrostate shows an H8 vertical to the membrane, alike an extended loop from TM7. The hook shape of H8 with TM3 is possible to form a new binding site (Fig. 6B). The active-like macrostate is overall similar to the crystal structure, but its TM6 shows an increasing open conformation (Fig. 6C).

Besides the distinctions around the conformation-describing parameters, the ligand pocket also shows differences. The different contact profiles in the inactive-like and active-like macrostates show the movement of TM6 in the active-like one (Fig. 6A and C). It is noteworthy that the intermediate state shows a different ligand binding mode, which shows an extended conformation towards the intracellular side and contacts with the toggle switch residue W269^6.48^ (Fig. 6B). The novel binding pose shows the plasticity of the AT_2_R orthosteric pocket and potential multiple signal pathways inside it.

### 3.4 The discovery of the potential cryptic allosteric site in the intermediate state

**Fig. 7:**
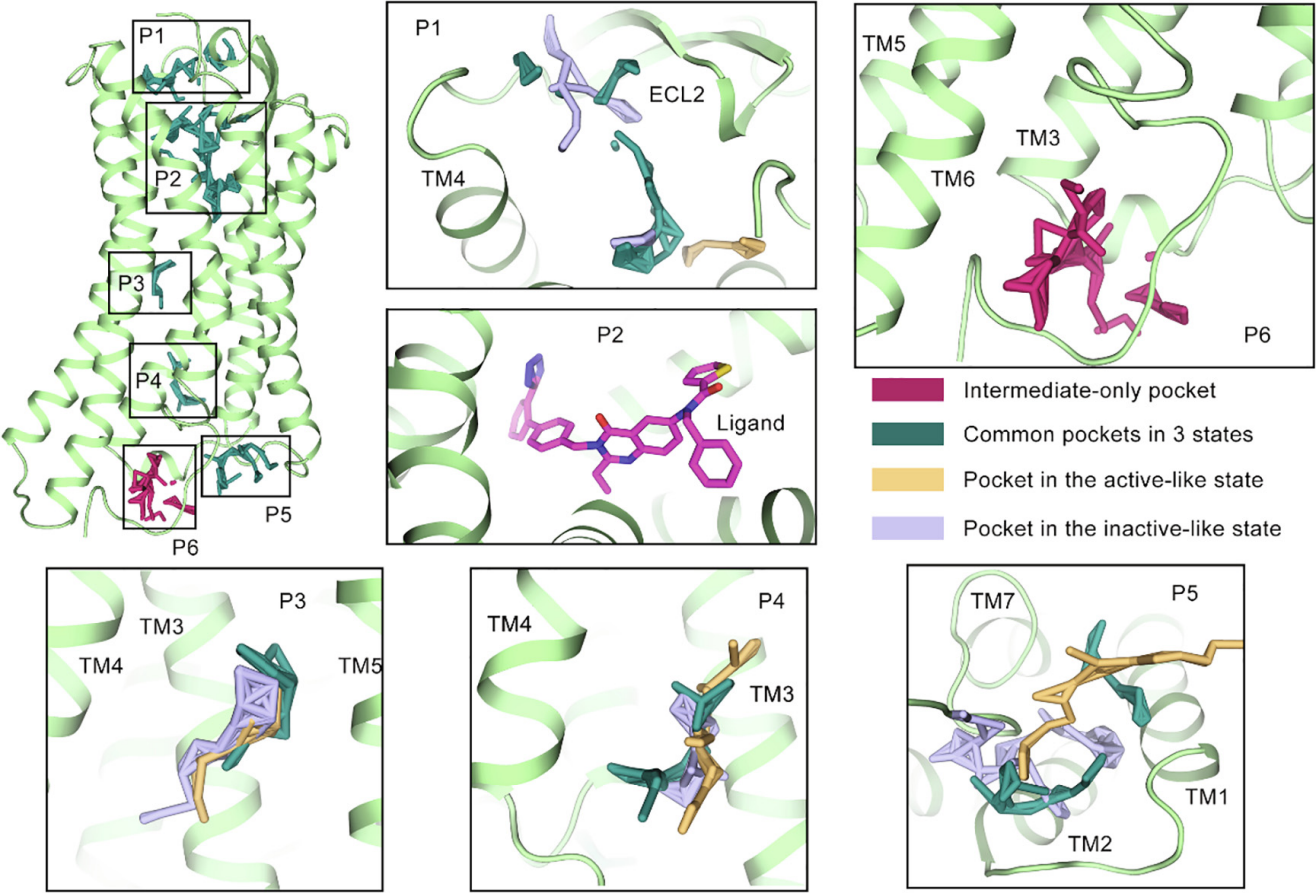
The pockets predicted by Fpocket in the intermediate state. Pockets are shown in sticks and zoom-in subplots show the specific position of each pocket. The pockets overlapping with those in other macrostates are colored in deep green, while the unique pocket P6 for the intermediate state was colored in red. Purple and orange sticks depict pockets in the representative inactive-like and active-like structures, respectively. (For interpretation of the references to color in this figure legend, the reader is referred to the web version of this article.)

We also detected potential pockets on the 3 macrostates to provide possible regulatory sites in AT_2_R. Using Fpocket [64], 6 pockets were identified in the intermediate state and P6 was shown as the sole pocket hidden in the state (Fig. 7). From the extracellular to the intracellular side, 6 pockets are distributed on the cavities of AT_2_R. P1 locates at the center of ECL2 and TM4, which overlaps with the site in the inactive-like macrostate. It is close to the LY2119620 allosteric pocket in the M2 muscarinic acetylcholine receptor (PDB 4MQT) [65]. Below it, P2 is the binding site of the antagonist. P3 situates at the membrane side among TM3-TM5, while P4 is at a lower position. The two pockets are also predicted in both two other macrostates. P5 locates at the intracellular side, the center of TM1, TM2, and TM7, which coincides with the vercirnon allosteric site in CCR9 (PDB 5LWE) [66]. Since the hook shape of H8 with TM6 is the uniquity of the intermediate state, P6 is the only pocket hidden in the intermediate state. Considering the large proportion of the intermediate state in the ensemble, the potential of P6 is worth noting.

**Fig. 8:**
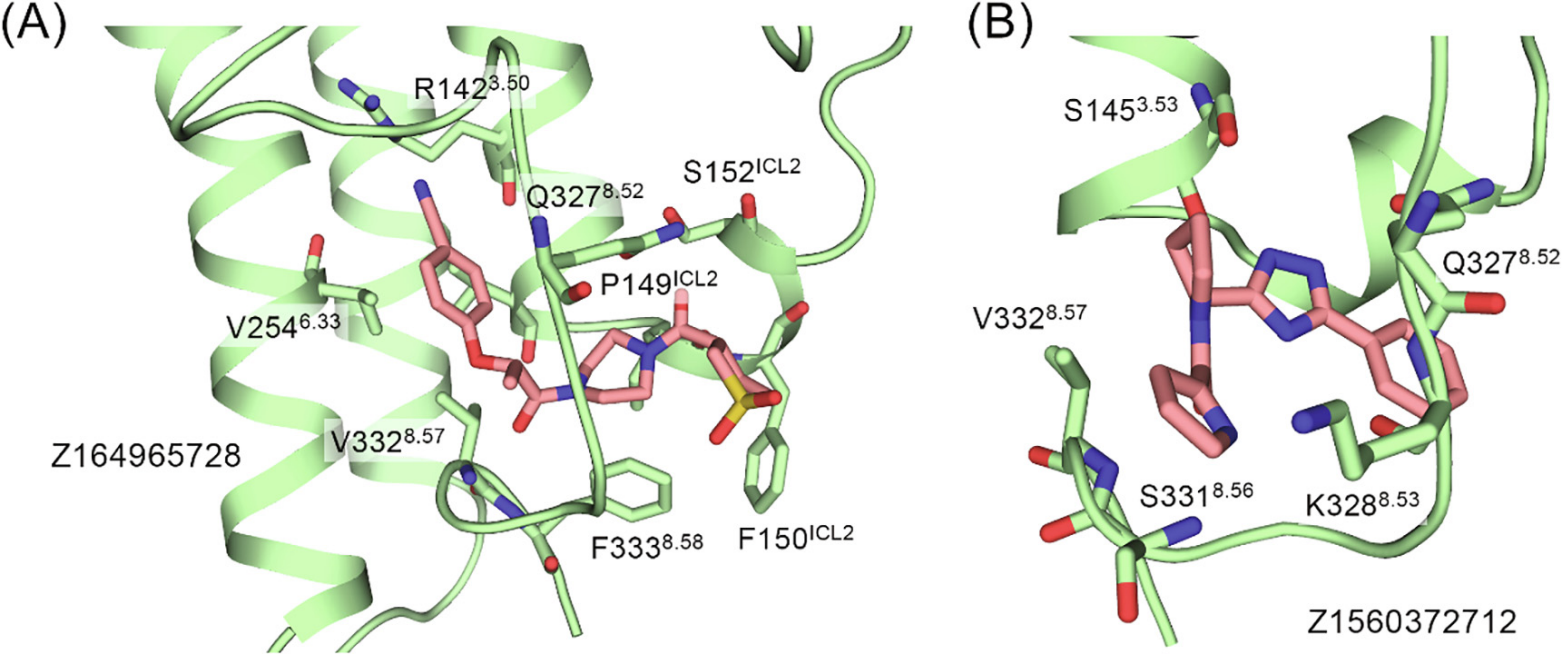
The two possible ligand binding modes for P6. (A) The docked pose for Z164965728, representing the “up” pose interacting with ICL2, TM3, TM6, and H8. (B) The docked pose for Z1560372712, representing the “down” pose mainly interacting with H8.

With the obtained P6, we applied virtual screening on it from the allosteric GPCR sublibrary of Enamine and different binding modes of potential ligands are shown in Fig. 8. The ligand conformations reflected two possible ways for the pocket to interact with regulators. One is the “up” conformation contacting with R142^3.50^ and intracellular TM6 residues (Fig. 8A), which shows a large binding interface and polar contact with R142^3.50^. Z164965728 is the representative ligand, whose binding is also stabilized with intensively hydrophobic contacts with V254^6.33^, F333^8.58^, and F150^ICL2^. The other is the “down” conformation constrained in the hook shape of H8 and interacting mainly with H8 residues (Fig. 8B). Z1450372712 shows a representative binding pose which is stabilized via polar contacts with S145^3.53^, S331^8.56^, and K328^8.53^. The two binding modes may provide insights into further structure-based drug design on P6.

## 4 Discussion

Captured by our all-atom unbiased MD simulations, the conformational space of AT_2_R shows a transition pathway from active-like to inactive-like states. With MSM, the macrostate distribution reflects that the stable inactive-like structures show a larger dihedral, indicating an increasing inward movement of H8 towards the TM bundle center. The active-like macrostate also shows a broader distribution than the static crystal structure. Combined with an evolutional algorithm and structure-based prediction methods [67], [68], [69], [70], the stabilized H8 position in MD simulation may provide a potential site for downstream factors, thereby offering insights into the search for the downstream pathway of AT_2_R.

Compared with the conformational ensemble in its homolog AT_1_R without ligand [28], antagonist-bound AT_2_R shows fewer active-like state conformations and a shorter transition time between different states. It infers that the antagonist disfavors the active-like conformation and accelerates the transition between states. Considering the 500 ns length of our simulation, many snapshots during the transition are defined as their original states. Hence, we did not observe a very small number of active-like state despite the antagonist. However, the transition time has been captured to favorite non-active states, inferring a biased distribution towards inactive state upon enough simulation time.

Several ATIPs have been discovered to interact with the H8 and C-terminal of active AT_2_R [71], [72]. From the conformational ensemble of AT_2_R, the interface for AT_2_R and ATIP is hidden in the TM bundle center in the inactive state, preventing AT_2_R from interactions. Upon activation, the interface residues have various conformations as shown by the dihedral in the free energy landscape. Since the active-like structure has half of H8 covered by the membrane, the interface residues may not be able to bind with ATIP in the state. Hence, the interactions with ATIP may involve a metastable intermediate state during the activation process and the state could be stabilized by ATIP.

From our dynamical and evolutional information, the signal transferring inside AT_2_R is overall similar to normal class A GPCRs. For example, the red sector in SCA shows the relationship between the ligand site and intracellular TM5-H8. Moreover, DCCM also infers the correlation of movement from extracellular to intracellular sides. But subtle differences still exist. The weak connection between the orthosteric site and classical downstream protein site reflects that AT_2_R is not dependent on the classical signal pathway. Considering that AT_2_R is controversial to interact with classical downstream proteins, the remains of the normal class A GPCR pathway may not perform as well as other GPCRs.

The DCCM matrix identified TM1, ICL1, and TM2 as the structures highly related to the movement of H8, inferring the specific conformational ensemble of H8 correlate with TM1-TM2 sequences. Also, experimental evidence has shown that the specific sequence in AT_2_R ICL1 leads to the observed atypical conformation of H8 [73]. Additionally, ICL3 has been identified as the key domain for initiating downstream signals of AT_2_R [74], [75]. In our *in silico* results, it shows evident movement in the decomposition of PCA and the free energy landscape. It reflects that ICL3 is a structural component with obvious dynamics, probably related to its function in signal initiating.

Since a potential pocket P6 has been identified in the hidden intermediate state, the regulation of AT_2_R may be benefits from the drug developed targeting P6. The position is always considered as the protein-protein interface in class A GPCRs and the potential of developing small molecule ligands targeting the interface has been proved [76], [77], [78]. In addition, the P6 site tends to be an allosteric one since classical downstream proteins bound here do not interact with AT_2_R [8], [79], [80]. Hence, regulators targeting P6 may show greater selectivity and less toxicity as the common advantage for allosteric modulators [81], [82], [83], [84], [85], [86]. Notably, the result of virtual screening are shown for possible ligand binding mode for P6 but their bioactivity was not confirmed by experiments.

## 5 Conclusion

Here, the all-atom unbiased MD simulations completely show the conformational space of AT_2_R and dynamically explained the transitions between different macrostates. With evolutional insights, the dynamic analysis provides the difference of AT_2_R compared with other class A GPCRs, thereby suggesting the attribution of its unique conformational space. The cryptic pocket hidden in the intermediate state also offers an opportunity for the development of novel AT_2_R regulators. Overall, our research elucidated the dynamic properties of AT_2_R and aids in the explanation of its unique mechanism.

## Declaration of Competing Interest

The authors declare that they have no known competing financial interests or personal relationships that could have appeared to influence the work reported in this paper.
